# Specific Cues Associated With Honey Bee Social Defence against *Varroa destructor* Infested Brood

**DOI:** 10.1038/srep25444

**Published:** 2016-05-03

**Authors:** Fanny Mondet, Seo Hyun Kim, Joachim R. de Miranda, Dominique Beslay, Yves Le Conte, Alison R. Mercer

**Affiliations:** 1 INRA, UR 406 Abeilles et Environnement, 84914 Avignon, France; 2 Department of Zoology, University of Otago, Dunedin 9054, New Zealand; 3 Department of Ecology, Swedish University of Agricultural Sciences, 750-07 Uppsala, Sweden

## Abstract

Social immunity forms an essential part of the defence repertoire of social insects. In response to infestation by the parasitic mite *Varroa destructor* and its associated viruses, honey bees (*Apis mellifera* L.) have developed a specific behaviour (varroa-sensitive hygiene, or VSH) that helps protect the colony from this parasite. Brood cells heavily infested with mites are uncapped, the brood killed, and the cell contents removed. For this extreme sacrifice to be beneficial to the colony, the targeting of parasitized brood for removal must be accurate and selective. Here we show that varroa-infested brood produce uniquely identifiable cues that could be used by VSH-performing bees to identify with high specificity which brood cells to sacrifice. This selective elimination of mite-infested brood is a disease resistance strategy analogous to programmed cell death, where young bees likely to be highly dysfunctional as adults are sacrificed for the greater good of the colony.

Honey bees (*Apis mellifera* L.) are cavity-nesting eusocial insects living in large colonies of around 40,000 sterile workers, a single fertile queen and, during the reproductive season, a few thousand males. Colony integrity is maintained through social interactions involving a range of sensory signals, in particular olfactory cues. Kin recognition and assessment of nestmate health-status are central to these social defence mechanisms^1^,^2^. Large numbers of individuals acting cooperatively is central to the success and survival of honey bees (*Apis mellifera* L.), but a high density of individuals and extensive social interactions between nestmates, make honey bee colonies innately susceptible to disease epidemics. To control disease spread, honey bees have developed behavioural responses to pathogens that provide social immunity, which complements the individual immune defences mediated by inducible molecular agents and microbial symbionts^3^,^4^. One of the most important social immunity defences is hygienic behaviour, which involves the detection and subsequent removal of abnormal or diseased brood^5^. Hygienic behaviour is particularly effective during the open brood phase, when eggs or larvae are easily accessible for inspection. Once brood cells are capped, the detection of abnormalities in developing (pre)-pupae becomes much more difficult. One consequence of this is that there is often an inverse relationship between the virulence of brood pathogens at individual level versus colony level, as pathogens adjust their virulence either to escape detection during the open brood phase^6^ or to ensure completion of the capped brood phase and emergence of the (infected) young adults^7^. Social immune management of the capped brood phase therefore acquires special significance, as it represents a potential haven for pathogens and parasites seeking to avoid detection and removal elsewhere.

This is the context that has allowed the recently acquired, exotic parasite *Varroa destructor* to become the main pathogenic threat facing honey bee colony survival worldwide^8^. The mite reproduces on developing pupae during the capped-brood phase, transmitting as it does so several virus species, particularly members of the *Deformed wing virus* (DWV) and *Acute bee paralysis virus* (ABPV) species complexes^9^,^10^. Although the mite also transmits viruses to adult bees, it is transmission during the pupal phase that is the most damaging to the colony, as this results in very short-lived and dysfunctional adult bees, thus severely disrupting the social organization and demographic continuity of the colony^7^. In the absence of control against this mite, parasitized western honey bee (*Apis mellifera* L.) colonies generally collapse within 2–3 years of infestation^11^. This contrasts strongly with the stable host-parasite equilibrium that varroa has with its original host, the Asian honey bee *Apis cerana*: an equilibrium that is largely maintained by an efficient set of unique social defence mechanisms^8^. Nonetheless, there are some *A. mellifera* populations that naturally survive varroa infestation without treatment^12^,^13^,^14^, implying that the combined social and individual defence mechanisms of *A. mellifera* are sufficiently plastic to adapt to new threats such as varroa. One such adapted social defence is varroa-sensitive hygiene (VSH), which involves the specific detection and removal of varroa-infested (pre)-pupae^15^. This slows the varroa population growth, increases colony survival^3^ and has been identified as one of the key components for varroa resistance^15^. Evidence suggests that VSH involves chemical communication^16^,^17^,^18^, but how adult bees choose which (pre)-pupae to sacrifice remains a mystery, as does the nature of the trade-off between the sacrifice of parasitized individuals and the growth of the colony as a whole.

By comparing the characteristics of individual brood targeted for removal by VSH behaviour (TA) with those of age-matched brood from adjacent varroa-infested but non-targeted (NT) cells and non-infested (NI) cells, this study suggests how adult bees choose which (pre)-pupae to sacrifice.

## Results and Discussion

### Selective targeting of impaired brood

**Figure 1:**
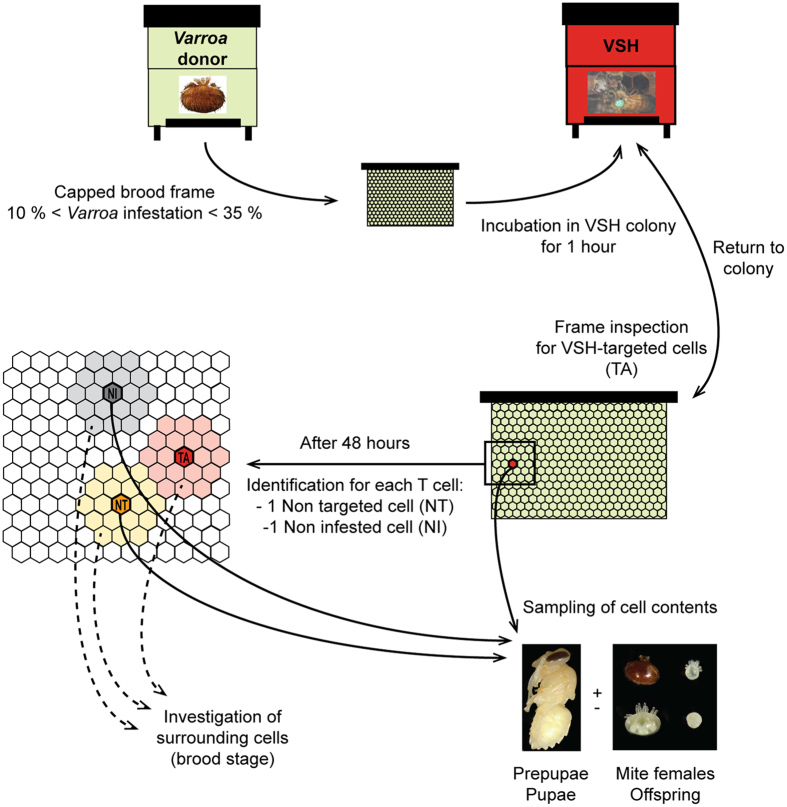
Schematic representation illustrating the design of the behavioural assay. Through the use of varroa-donor frames, brood cells that were varroa-parasitized and VSH-targeted (TA, targeted – red), varroa-parasitized and non-targeted (NT, non-targeted – orange) and non-parasitized (NI, non-infested – grey) were collected.

The VSH behaviour observed in this study was performed by healthy bees on highly varroa-infested brood frames. Four putative VSH-triggering signals were assessed: mite numbers, developmental stage of sampled bees relative to brood in the immediately surrounding cells, virus infection, and brood pheromone levels. A summary of the experimental design used in this study is shown in Fig. 1.

**Figure 2:**
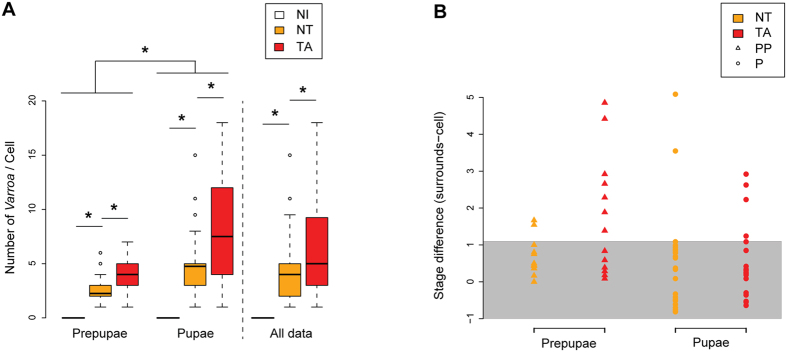
Brood and mite content of the sampled capped cells, and their surrounding cells. (**a**) Size of the mite families (females and offspring). NI, non-infested; NT, non-targeted; TA, targeted. Significant differences are indicated with asterisks (LMEM, 95% confidence intervals, 15 ≤ n ≤ 42). (**b**) Delay in development, as estimated by the differences between the developmental stage of brood sampled in each cell type, and the average developmental stage of brood collected from surrounding cells. The grey zone indicates a difference ≤ 1 developmental stage.

Targeted (TA) cells had significantly more mite family members than non-targeted (NT) cells (Fig. 2A: LMEM: t = 4.327, CI_95%_ = [1.348, 3.581]), confirming earlier studies relating VSH behaviour to the number of mites in the brood cell^19^, the infesting mite’s reproductive success^15^,^20^, and the removal of developing bees likely to cause the greatest damage to the colony in terms of social breakdown and pathogen spread^18^. VSH behaviour was also heavily directed towards pre-pupae and mid-stage P5 pupae (30% each), with the remainder spread among the other pupal stages (see Supplementary Fig. 1).

The difference in developmental stage between the sampled brood cell and its immediate neighbours was used as a marker for developmental delay: a possible trigger for removal by VSH. Most of the non-targeted, infested cells were within one developmental stage of their nearest neighbours (Fig. 2B). By contrast, about one third of VSH-targeted pupae were up to 3 developmental stages delayed. The difference was even greater for targeted pre-pupae, of whom about half were up to 5 developmental stages delayed (Fig. 2B). This is the first evidence that VSH behaviour is selective towards developmentally delayed brood, brood that are likely to become dysfunctional adults if allowed to emerge.

### Abnormal brood ester pheromone profiles are targeted

**Figure 3:**
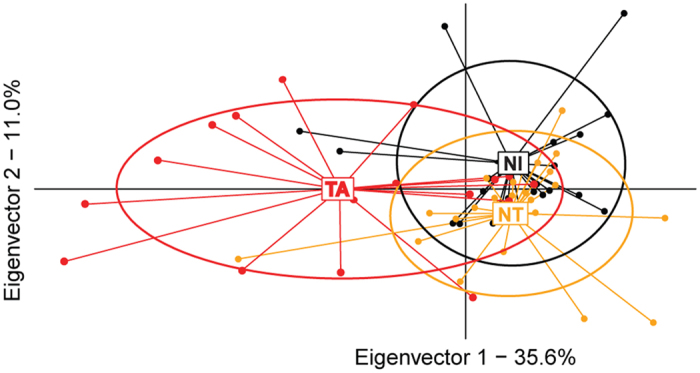
Quantitative analyses of brood ester pheromone (BEP) profiles. Discriminant analysis of VSH-targeted (TA, red), non-targeted (NT, orange) and non-infested (NI, black) cells, based on the 9 BEP compounds that were detected in the samples (18 ≤ n ≤ 24 per group). Ellipses cover 67% of the samples of each group.

Such developmental delays point to a breakdown of the normal metamorphosis process, which is guided by precisely timed hormonal signals. Brood produces brood ester pheromone (BEP), a blend of ten ethyl and methyl esters that impact the physiology and behaviour of honey workers^21^,^22^. Brood in capped cells usually release low amounts of BEP^23^, and deviations in the composition or amount of BEP could be sufficient to trigger VSH behaviour^24^,^25^,^26^. To examine this possibility, BEP profiles were examined in brood from TA, NT and NI cells. Discriminant analysis revealed differences in amounts of the 10 BEP components in brood from TA cells and NT/NI cells (Fig. 3A - λ_Wilks_ = 0.6, F_14,110_ = 2.29, p = 0.0087, 17 ≤ n ≤ 23), and comparison of paired Mahalanobis distances between the BEP profiles of VSH-targeted and non-targeted/non-infested brood confirmed that targeted brood produce a distinctive BEP profile (see Supplementary Table 1). Quantitative analysis indicates moreover that this difference in BEP profile is primarily accounted for by deviations in BEP production of pupae, and not of pre-pupae: pupae from TA cells produced a significantly higher amount of BEP than pupae from NT cells (LMEM: t = 2.935, CI_95%_ = [69.464, 348.796]). This difference represents an increase of 130% in brood pheromone released by TA pupae as compared to NT pupae. Bees respond differently to BEP depending on the concentration and component ratios of the pheromone^21^. It has also been established that the responses to individual components of this pheromone are context dependent^22^. Because capped cells normally release small amounts of BEP^23^, it is likely that a capped cell with high levels of BEP will be interpreted as “abnormal”. Whether the unique BEP profile of these targeted pupae is a causative signal for removal by bees VSH-performing bees is currently being investigated.

### Targeting of KBV-infected bees

One possible reason for the physiological impairments leading to abnormal chemical profiles are virus infections transmitted by varroa during reproduction in the pupal phase^18^. The principal viruses transmitted by varroa are those of the DWV and ABPV virus complexes^9^,^10^, whose representatives in New Zealand are DWV and Kashmir bee virus (KBV) respectively^27^.

**Figure 4:**
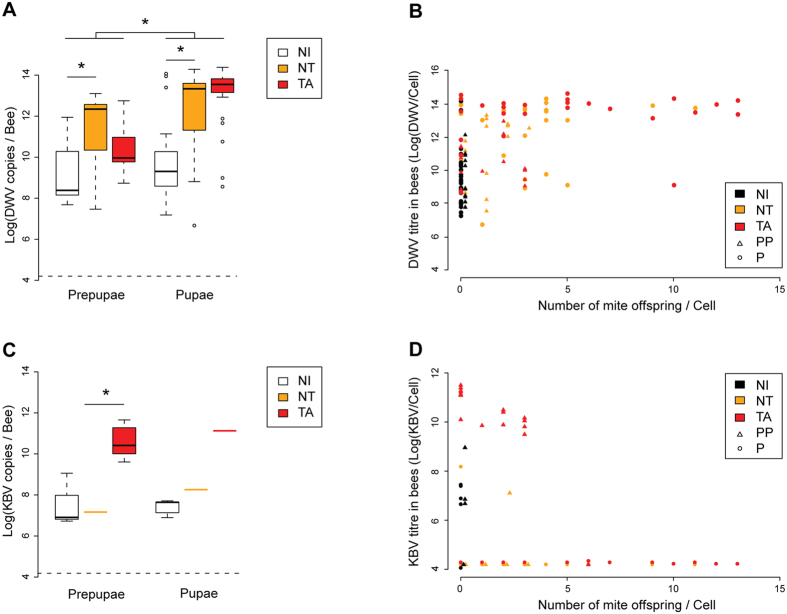
Virus titres in capped brood cells. (**a**) DWV or (**c**) KBV titres in pre-pupae and pupae from VSH-targeted (TA), non-targeted (NT) and non-infested (NI) cells (Log_10_ virus copies/bee). Asterisks indicate significant differences (LMEM, 95% confidence interval, n = 42 for each group). (**b**) DWV or (**d**) KBV titres in bees from TA, NT and NI cells versus the number of mite offspring found in each brood cell. TA pre-pupae clearly stand out from the other brood cells in terms of KBV infection.

We found a significant difference in DWV titre between varroa-infested cells and uninfested cells (as expected), but not between targeted and non-targeted cells containing brood at either pre-pupal or pupal stages (Fig. 4A,B - LMEM: t = 1.490, CI_95%_ = [−0.227, 1.680]). This contrasts sharply with KBV-infected cells, which were effectively and specifically targeted by VSH behaviour at the pre-pupal stage (Fig. 4C,D - LMEM: t = 6.770, CI_95%_ = [2.904, 5.271]).

Apart from illustrating the different virological effects of ABPV complex (i.e. KBV) and DWV on (pre)-pupae, these results show that at least part of the VSH behaviour is disease-specific, and involves multiple cues. Pre-pupal targeting is associated with KBV infection but is signalled through cues other than the BEP profile. Pupal targeting is associated with BEP abnormalities, though not necessarily from DWV infection. The results also imply that infested brood has a much better chance of escaping VSH detection with DWV infection than with KBV infection, thereby providing a mechanistic explanation for both the gradual disappearance of ABPV complex (i.e. KBV) and the long-term persistence of DWV in newly varroa-infested colonies^7^,^27^. Interestingly, the study identifies a positive role for virus infections in bee social health by encouraging the early detection and removal of varroa-infested brood, a strategy for disease resistance analogous to programmed cell-death and the hyper-sensitive response^28^. The significance of this viral contribution is illustrated by greatly increased VSH efficiency when accompanied by high virus titres^18^. However, DWV, even at high titres, is only partly effective in triggering VSH^18^, which allows mite-DWV epidemics to break through the colony’s social defences and kill the colony. A significant additional factor is that VSH behaviour appears also to be dependent on the sensory acuity of the detecting bees, which may be compromised^29^ by the same viruses facilitating the detection of varroa-infested brood. Thus, VSH behaviour itself may also break down through varroa-transmitted virus epidemics, placing limits on its ability to control mite infestation. Avoiding this breakdown is therefore critical to colony survival.

This study investigates a long-standing question central to the success and survival of honey bee colonies infested with varroa: how do the worker bees that perform VSH behaviour determine which brood to select and sacrifice? Here we show that unique signatures clearly differentiate targeted from non-targeted bees. Bees sacrificed display either, extreme levels of KBV infection, which is typical for targeted pre-pupae, or profoundly abnormal pheromonal bouquets. As these bees are predicted to be highly dysfunctional, their removal will have minimal impact on the colony workforce. However, by sacrificing these siblings, VSH bees disrupt the mite during its reproductive cycle and reduce its reproductive success. Our findings suggest two testable hypotheses: first, that VSH is triggered by unusual pupal BEP profiles specifically associated with varroa-infested brood and second, that these altered BEP profiles are due to developmental delays caused by specific varroa-transmitted virus infections. Work is underway to test these hypotheses.

## Materials and Methods

### Honey bee colonies and behavioural assay set up

This study was performed using *Apis mellifera* L. honey bee colonies located at the Department of Zoology of the University of Otago (New Zealand), from February to April 2014. Virus quantification was undertaken in the University of Otago Zoology Department, and chemical experiments were conducted at the INRA (National Institute for Agricultural Research) research centre of Avignon, UR 406 Abeilles et Environnement, France.

A behavioural assay was designed to enable the contents of capped brood cells selectively targeted by VSH workers to be sampled (Fig. 1). Two colonies, selected on the basis of their strong hygienic response against varroa (BettaBees Ltd, New Zealand, unpublished data) were named “VSH” colonies (Fig. 1). In addition, several apiaries were screened to select colonies exhibiting varroa (*Varroa destructor*, Korean haplotype) infestation in 10 to 35% of their brood. These colonies are referred to here as “varroa donor” colonies (Fig. 1).

Every two days, a naturally mite-infested frame containing capped brood was removed from a “varroa donor” colony and placed in the centre of the brood nest of a “VSH” colony. The precise location of any uncapped cell, or cell with an opening in the cap, was recorded on a transparent plastic sheet placed over the frame during an initial frame inspection. After 1 hour of incubation in the “VSH” colony, the frame was removed and brought back to the laboratory. Newly opened and/or uncapped brood cells were identified and marked on the transparent sheet. Cells that only showed a tiny hole, in which the pupa had already been eaten, or in which the pre-pupa/pupa was dead, were discarded from the experiment. This latter criterion was set to avoid any possible confusion between removal cues originating from dead brood and cues for removal originating from varroa-parasitised brood^30^. VSH-targeted cells were coded as TA cells (Fig. 1). The frame was then placed back in the “VSH” colony for another hour, before being resampled.

After 48 hours of observations, each sampling frame was brought back to the laboratory. The location of all TA cells was recalled using the transparent plastic sheet. For each TA cell, a capped cell located 2 to 6 cells away that was also varroa-parasitised and that contained a developing bee at the same stage of development as that found in the TA cell, was selected randomly. Such cells, despite having a similar potential to be VSH-targeted had not been selected by the worker bees over the course of the 48 h observations. These non-targeted cells were considered as negative controls and named NT cells (Fig. 1). Similarly, another cell, also 2 to 6 cells away from the TA cell, but one that was not varroa-parasitised was randomly selected and used as a second negative control. These cells were named non-infested cells (NI – Fig. 1).

### Sample collection

For each VSH-targeted cell (TA) and control cell (NT, NI), the pre-pupa/pupa as well as the mite family were carefully removed using soft forceps and a fine brush, respectively. The stage of the developing bee and the composition of the mite family (if the cell was infested) were recorded^31^. For chemistry analysis, the contents of 4 cells ((pre)-pupae and/or mite families) were sequentially placed in a 4 mL glass tube, weighed and stored at −30 °C until further processing. For virus analyses, (pre)-pupae and corresponding mite families were separated and individually placed in a 5 mL plastic CryoS tube (Greier Bio One), and immediately flash frozen in liquid nitrogen before being stored at −80 °C until further processing.

In total, 450 samples were collected for further analysis of virus content or chemical profiles, with 60 cell-content samples (bees and mites), 126 bee (pre-pupae and pupae) samples and 84 mite samples.

In addition, the surroundings of each sampled cell were investigated, by opening the 2 rings of capped cells closest to the sampled cell (Fig. 1). In each cell of the surroundings, the stage of the developing bee as well as the composition of the mite family (if the cell was infested) were recorded.

### Chemical analyses of the brood ester pheromone (BEP) profiles

#### Extracts preparation

The BEP compounds were extracted by crushing 4 individuals (pre-pupae or pupae) in 1900 μL of hexane (Sigma Aldrich) and 100 μL of C17 ester methyl heptadecanoate (10 ng/μL; internal standard), using self-made circular glass pestles. Crushed samples were immediately placed on ice, and stored at −30 °C. The tubes were then centrifuged at 4,000 rcf at 4 °C for 20 min. The supernatant was transferred to new tubes and stored at −30 °C until fractionation.

Sample extracts were applied to a silica column (silica gel 60, particle size 40–63 mm, 230–400 mesh). The column was first rinsed with a solvent mix (98.5% isohexane, 1.5% diethyl ether – Sigma-Aldrich) until 3 mL of the mix was collected. Samples were added to the column and eluted in a final volume of 3 mL of the solvent mix containing 1.5% of diethyl ether. The second fraction was eluted in a final volume of 3 mL of a second solvent mix (94% isohexane, 6% diethyl ether). The 3 mL of the second fraction, containing the BEP compounds, were concentrated to 30 μL under a nitrogen stream.

#### Gas chromatography

Quantitative analysis of the BEP compounds contained in the extracts of the second fraction were performed on a fast gas chromatograph (Shimazu GC-2014) equipped with a split/splitless inlet, a flame ionization detector, and a capillary Supelcowax column (15 m × 0.10 mm, 0.10 μm film thickness). 1 μL of each sample was injected with a 30-ratio split mode, and a column flow of 0.94 mL/min. Carrier gas was hydrogen and temperatures of the injector and detector were both set at 250 °C. The oven temperature was programmed with the following conditions: 90 °C isothermal held for 1 min, followed by temperature increases at a rate of 40 °C/min up to 195 °C, at a rate of 1 °C/min up to 201 °C, and at a rate of 40 °C/min up to 250 °C. The oven was finally held at 250 °C for 3 min. The peaks corresponding to the internal standard and each compound of the BEP were attributed by injection of pure commercial compounds (Sigma Aldrich) diluted in isohexane at the end of each batch of 10 samples.

Quantification was obtained using a standard curve constructed for each compound of the BEP, and normalised by the internal standard concentration of each sample, after correction of the slope differences between the BEP standard curve and the C17 ester standard curve. Between 17 and 23 samples of each category (NI, NT, TA) were analysed.

#### Mass spectrometry

Confirmation of the identity of the BEP compounds in the samples was performed on a mass spectrometer (ThermoQuest Trace GC - Polaris) equipped with a Phenomenex ZB-WAX column (30 m × 0.25 mm, 0.25 μm film thickness). 1 μL of a selection of 8 representative samples was injected with the inlet in splitless mode, and a column flow of 0.7 mL/min. Carrier gas was helium and temperatures of the injector and detector were set at 250 °C. The oven temperature was programmed with the following conditions: 60 °C isothermal held for 1 min, followed by temperature increases at a rate of 30 °C/min up to 180 °C, at a rate of 1.5 °C/min up to 210 °C, and at a rate of 20 °C/min up to 250 °C. The oven was finally held at 250 °C for 5 min. Identity of the compounds was confirmed by comparison of their mass spectra to those available in libraries, and further comparisons of their retention indices.

### Virus quantification analyses

#### RNA extraction of bee samples

Samples of pre-pupae and pupae were individually crushed for 30 sec in 500 μL of GITC buffer using a grinding probe (Ultra Turrax T25 – IKA Labortechnik). Total RNA was extracted from 100 μL of each homogenate following the RNeasy plant mini kit protocol (Qiagen), eluted in 50 μL nuclease-free water and stored at −80 °C until further processing. Within each batch of 20–30 samples, one “blank” extraction was performed using only GITC buffer, to test for contamination. RNA yield, concentration and quality were measured using a NanoDrop ND-100 spectrophotometer (NanoDrop Technologies).

#### RNA extraction of mite samples

Mite family samples were individually crushed for 30 s in 500 μL of trizol and 5 μL of carrier RNA (5 ng/μL – Invitrogen). Samples were incubated for 5 min at RT and after transfer into 1.5 mL Eppendorfs, 100 μL of chloroform was added. Samples were hand-shaken for 15 s and let set for 3 min at RT. They were then centrifuged at 12,000 rcf for 15 min at 4 °C. The upper phase was gently pipetted out. Total RNA was extracted following the Purelink Micro kit (Invitrogen), eluted in 10 μL nuclease-free water and stored at −80 °C until further processing. Within each batch of 20–30 samples, one “blank” extraction was performed using only trizol, to test for contamination. RNA yield, concentration and quality were measured using a NanoDrop ND-100 spectrophotometer (NanoDrop Technologies).

#### cDNA synthesis

For each sample, 150 ng of total RNA was reverse-transcribed in 10-μL reaction volumes using random hexamer primers and the Superscript III VILO cDNA synthesis kit (Invitrogen), according to the manufacturer’s protocol. Each reaction also contained 0.1 ng of synthetic RNA250 (Ambion), added to the reaction mix as a passive reference gene for evaluating the cDNA reaction efficiency for each RNA sample^32^.

#### qPCR assays and data conversion

Real-time qPCR was performed using primers designed to detect three honey bee viruses: deformed wing virus (DWV), Kashmir bee virus (KBV) and sacbrood virus (SBV). DWV and KBV were chosen for their high prevalence, amount and virulence in (pre)-pupae^9^,^10^. Because of the extremely low prevalence of KBV in pupal samples, quantitative analysis could only be performed on KBV titres in pre-pupae. Sacbrood virus (SBV) presence was also investigated. This virus affects mainly the larval stage and it has not been directly linked with varroa parasitism^33^. However, several of the VSH-targeted pre-pupae observed in this study displayed SBV-like symptoms (e.g. brownish colour, sac body shape). SBV was included because we wanted to selectively investigate cues for VSH and not for any other type of hygienic behaviour that can develop in response to moribund (pre)-pupae suffering from many different stressors. No single sample was detected positive for SBV. This result confirmed that our samples are representative of stress created by varroa. This “varroa specificity” was also insured by not including in the analysis any individuals that appeared dead. Therefore, several pre-pupae were excluded from the analysis in order to exclude any risk of confusion between mortality and “varroa-infestation” cues for removal. This difference is at the basis of the distinction between VSH behaviour and standard hygienic behaviour^30^,^34^.

Each sample was also assayed for the passive reference RNA250. The assay primers and performance parameters are given in Supplementary Table 2^35^,^36^. All samples were run in duplicate.

The assays were run on a Mx3000P thermocycler (STRATAGENE) using Express SYBR GreenER qPCR SuperMix (Invitrogen) in 20-μL reaction volumes containing 3 μL of 1:10 diluted cDNA template, and 0.2 μM of each primer.

The cycling parameters were an initial denaturation step at 95 °C for 2 min, followed by 40 cycles of denaturation for 15 s at 95 °C, annealing for 20 s at 58 °C, and extension for 30 s at 72 °C followed by fluorescence reading. The amplification was followed by a dissociation curve analysis of the PCR products by raising the temperature from 72 °C to 95 °C, in 0.5 °C increments.

Positive controls and non-template controls were included on each plate. Plasmids of known concentration containing inserts for each target were used to generate external standards for absolute quantification, obtained from 10-fold serial dilutions. Each plate contained at least 4 different concentrations of each external standard covering 7 orders of magnitude.

The specificity of each PCR product was verified using melting curve analysis and electrophoresis. Samples were assigned positive for a target if their melting temperature was similar to the melting temperature of the positive controls and if they had a Cq value no greater than 35.

The Cq values were determined at the same fluorescence threshold for all plates and all targets. For each target RNA, the Cq values of the external dilution standards of all RT-qPCR runs were pooled and plotted against their corresponding log_10_[template]. The linear regression equations were used to estimate the absolute amounts of virus and RNA250 RNA in each reaction. The regression slopes were used to calculate the amplification efficiencies (E) of the different assays using the following equation: *E*_*assay*_* = 10−*^*1/slope *^37 (see Supplementary Table 2).

For all positive samples, the absolute virus RNA abundances per cell were then calculated by averaging the Cq values of the duplicates and by multiplying the amount per reaction by the different reaction and extraction dilution factors, including the individual cDNA synthesis efficiency obtained through the RNA250 passive reference gene assay.

Virus titres were log transformed to account for the exponential distribution of the data. Because it is not possible to log transform zero values, samples considered as negative were assigned a hypothetical Cq value of 36, which was converted to theoretical virus titres as described above. These “negative virus titres” were averaged to obtain the titre detection threshold for each target.

Virus prevalence was defined as the percentage of cell contents (bees) displaying Cq values ≤ 35 for each viral target (bees: n = 126). Virus titres were calculated as presented above, and analysed on positive samples only (DWV_bees_: n = 126; KBV_bees_: n = 25).

### Statistical analysis

All statistical analyses and figures were generated in the R environment (Version 3.0.2). In all assays, capped brood cells (pre-pupa/pupa, or mite family) were considered as the individual, unless otherwise stated.

Due to the nature of the experimental designs, quantitative analyses were performed using linear mixed-effect models (LMEM – package *lme4*). Virus titres and BEP compound amounts were analysed in relation to the brood category (NI, NT, TA). To account for the fact that sampled brood cells could belong to a same colony or to the same sample triplicate, the identity of cells amongst experimental colonies and the identity of varroa donor colonies were included as a random factor, along with the brood category as a fixed explanatory variable. The process of generating a P-value is not straightforward for LMEM^38^. Therefore we provided 95% confidence interval (CI_95%_) as a tool for assessing significance of the fixed effects^39^. LMEM t-values and 95% confidence intervals of all quantitative analyses are provided in Supplementary Table 1.

Discriminant function analysis (DFA) allowed us to assess the explanatory power of the BEP compound variables on the clustering of the samples according the brood category (NI, NT, TA). DFA was built using the functions *dudi.pca* and *discrimin* (Package *ade4*), after centering and scaling the data. Evaluation of the clustering was performed with Wilk’s Lambda; Mahalanobis distances between all pairwise groups were calculated as estimates of the distances between each group. P-values were adjusted for multiple comparisons using Bonferroni’s correction.

## Additional Information

**How to cite this article**: Mondet, F. *et al.* Specific Cues Associated With Honey Bee Social Defence against Varroa destructor Infested Brood. *Sci. Rep.* **6**, 25444; doi: 10.1038/srep25444 (2016).

## Supplementary Material

Supplementary Information
