# Supplementary material for: Specific Cues Associated With Honey Bee Social Defence against Varroa destructor Infested Brood
Source: Sci Rep. 2016 May 3;6:25444. doi: 10.1038/srep25444 (PMC4853723; doi:10.1038/srep25444)
Supplement: Supplementary Information [file srep25444-s1.pdf]

Specific Cues Associated With Honey Bee Social Defence against *Varroa destructor* Infested Brood

Fanny Mondet [1,2], Seo Hyun Kim [2], Joachim R. de Miranda [3], Dominique Beslay [1], Yves Le Conte [1] and Alison R. Mercer [2]

(1) INRA, UR 406 Abeilles et Environnement, 84914 Avignon, France

(2) Department of Zoology, University of Otago, Dunedin 9054, New Zealand

(3) Department of Ecology, Swedish University of Agricultural Sciences, 750-07 Uppsala, Sweden

Supporting information

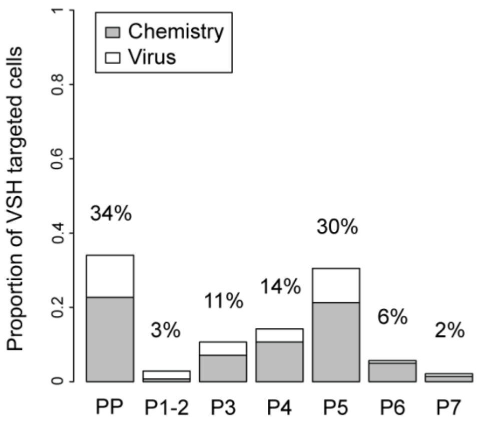

Figure S1. Frequency plot of the different stages of VSH-targeted developing bees.

PP, pre-pupae; P, pupae. Pupae were classified in different stages according to morphological characteristics.

Table S1. Pair-wise squared Mahalanobis distances between the BEP profile of non-infested (NI), non-targeted (NT) and VSH-targeted (TA) brood

	NI	NT
NT	D = 0.606<br>$F_{7,55}$ = 0.917<br>P = 0.5	
TA	D = 2.661<br>$F_{7,55}$ = 3.351<br>**P = 0.0048**	D = 2.461<br>$F_{7,55}$ = 3.154<br>**P = 0.0071**

P-values that are significant at the 5% probability after Bonferroni's correction are indicated in bold (P = 0.015).

Table S2. Primer sequences and performance indicators of the RT-qPCR assays run for the different honey bee viruses

Target	Primers	Sequence (5' – 3')	Size (bp)	E	r^2	Tm (°C)
DWV	DWV-F8688	GGTAAGCGATGGTTGTTTG	143	2.014	0.982	79.5
	DWV-B8794	CCGTGAATATAGTGTGAGG				
KBV	KBV-F6639	CCATACCTGCTGATAACC	200	1.870	0.995	82.5
	KIABPV-B6707	CTGAATAATACTGTGCGTATC				
SBV	SBV-qF3164	TTGGAACTACGCATTCTCTG	335	1.884	0.995	81.6
	SBV-qB3461	CTCTAACCTCGCATCAAC				
RNA250	RNA250-F	TGGTGCCTGGGCGGTAAAG	227	2.018	0.995	86.7
(Ambion)	RNA250-R	TGCGGGGACTCACTGGCTG				
